# Operational characteristics of a hospital rapid response team for public-area emergencies: a retrospective study

**DOI:** 10.3389/fmed.2026.1897760

**Published:** 2026-07-17

**Authors:** Shuihong Chen, Sa Wang, Qiaoli Chen, Yuwei Wang, Shuaishuai Zhou, Jiajun Ren, Chener Ye, Danping Yan

**Affiliations:** 1 Quality Management Office, The Second Affiliated Hospital Zhejiang University School of Medicine, Hangzhou, Zhejiang, China; 2 Department of Emergency Medicine, The Second Affiliated Hospital Zhejiang University School of Medicine, Hangzhou, Zhejiang, China; 3 Zhejiang Evaluation Center for Management Service and Administration, Hangzhou, Zhejiang, China

**Keywords:** hospital rapid response team, hospitals, non-inpatients, patient safety, public-area emergencies, workflow

## Abstract

**Background:**

A rapid response system (RRS) is widely used to identify and manage clinical deterioration in hospitalized patients. However, acute medical emergencies occurring in hospital public areas remain underexplored. This study aimed to describe the incidence pattern, clinical and operational characteristics of the RRS activation process, and factors associated with high-risk events among non-inpatient individuals in hospital public areas.

**Methods:**

This retrospective single-center study included all RRS activations involving non-inpatient individuals in hospital public areas at the main campus of the Second Affiliated Hospital, Zhejiang University School of Medicine, between January 2015 and December 2025. “Non-inpatient individuals” were defined as individuals who were not admitted to inpatient beds and were not under continuous bedside monitoring at the event location. Data regarding event location, trigger reason, activator identity, response time, on-site interventions, and patient outcomes were extracted from an intelligent emergency response information platform. Descriptive analyses were performed to evaluate temporal-spatial patterns and operational characteristics. Exploratory univariate analyses were conducted to examine factors associated with event severity.

**Results:**

A total of 217 RRS activations were included. Events occurred mainly in waiting areas (44.24%), outpatient clinical areas (29.49%), and medical examination areas (18.43%), with a clear daytime predominance. Nurses were the most frequent activators (61.75%). The median response time was 3.00 min. Common on-site interventions included oxygen therapy (25.8%), blood glucose monitoring (25.3%), cardiopulmonary resuscitation (11.5%), intravenous medication administration (10.6%), and bag-mask ventilation (9.2%). Most individuals were subsequently transferred to the emergency department (88.94%). Exploratory analyses suggested that older age and trigger reason were associated with a higher likelihood of high-risk events, whereas event location and time period were not significantly associated with severity.

**Conclusion:**

In this retrospective study of 217 public-area RRS activations, emergencies occurred predominantly in waiting areas, outpatient clinical areas, and medical examination areas, and most affected individuals required subsequent emergency department care. These findings suggest that a hospital-wide RRS can provide timely recognition and initial management for public-area emergencies involving non-inpatient individuals. Spatially distributed coverage, standardized response workflows, and digital process monitoring may contribute to improved emergency preparedness for non-inpatient individuals in hospital public areas.

## Introduction

In addition to inpatient wards, hospitals contain multiple public areas, such as outpatient clinics, waiting areas, diagnostic examination areas, corridors, elevators, and administrative spaces, where acute medical emergencies may occur among non-inpatient individuals, including outpatients, accompanying persons, hospital staff, and volunteers. Such events, including syncope, seizure, shock, respiratory distress, and cardiac arrest, often occur without warning and frequently involve individuals who are not under continuous monitoring (1–3).

A rapid response system (RRS) is widely used to identify and manage clinical deterioration in hospitalized patients and has been associated with earlier intervention and improved outcomes (1–3). However, evidence on the implementation of an RRS for non-inpatient individuals in hospital public areas remains limited, and operational challenges persist, including incomplete spatial coverage, non-standardized activation pathways, and insufficient evaluation of long-term system performance (4–7).

With the ongoing development of smart hospitals, digital information platforms, mobile data capture, and visual management tools offer new opportunities to improve emergency response workflows.

At our institution, the existing RRS was refined according to the principles of shortest route, nearest response, optimal allocation, and clear team accountability (8). An intelligent emergency response information platform was introduced to integrate event activation, structured data capture, visual monitoring, pathway analysis, and continuous quality improvement.

From an operational management perspective, RRS activations in hospital public areas are not only clinical rescue events but also important workflow nodes within hospital-wide emergency governance. These events involve early recognition by heterogeneous witnesses, rapid dispatch of response teams, on-site stabilization, and timely handoff to the emergency department or other definitive care pathways. Therefore, structured recording and digital monitoring of such events may help identify high-risk locations, clarify response responsibilities, optimize resource allocation, and support continuous improvement of emergency workflows.

However, real-world evidence regarding the incidence pattern, clinical characteristics, and operational performance of RRS activations involving non-inpatient individuals in hospital public areas remains limited. Therefore, the overall goal of this study was to provide evidence to support the optimization of hospital-wide emergency response for non-inpatient individuals in public areas. Specifically, using data from RRS activations involving non-inpatient individuals in hospital public areas between 2015 and 2025, this study aimed to describe the incidence and distribution of activations, the clinical and operational characteristics of the activation process, including event location, trigger reason, activator identity, response time, on-site interventions, and patient disposition, and to explore factors associated with high-risk events.

## Materials and methods

### Study design and setting

This retrospective single-center study included all RRS activations involving non-inpatient individuals in hospital public areas of the main campus between January 2015 and December 2025. For this study, “non-inpatient individuals” was used as an operational rather than diagnostic category and referred to persons who had not been admitted to an inpatient bed and were not under continuous bedside monitoring at the event location. This category included outpatients, accompanying persons, hospital staff, and volunteers. Because the operational database did not always distinguish whether some patient cases had completed emergency department registration but were still awaiting formal assessment, the cohort should be interpreted as describing public-area emergencies involving non-inpatient individuals and outside continuous monitored care rather than a strictly homogeneous patient population. Hospitalized patients and records with >20% missing data were excluded. All data were anonymized before analysis.

### RRS organization and activation

The hospital has operated a 24-h, 7-days-per-week RRS since 2012 (9). Coverage is organized according to building distribution and the shortest travel distance, with designated responsibility zones. The emergency response team includes second-line emergency physicians, ICU physicians, anesthesiologists, emergency nurses, and ICU nurses. Public areas are primarily covered by the emergency department second-line physician and emergency nurse.

Activation criteria were developed with reference to the National Early Warning Score (NEWS) (10). If any trigger criterion is met, any witness may activate the system by calling the in-hospital emergency hotline via mobile or fixed telephone or by pressing an emergency alarm button installed in public areas.

In this study, RRS activation referred to the hospital-wide rapid response pathway for acute medical events involving non-inpatient individuals in hospital public areas. It should be distinguished from code blue activation. In many healthcare systems, code blue is used specifically for suspected or confirmed cardiopulmonary arrest requiring immediate resuscitation. By contrast, the RRS pathway described in this study was broader and could be activated for a range of acute events, including sudden loss of consciousness, witnessed collapse, syncope or dizziness, seizure-like activity, allergic reactions, respiratory distress, gastrointestinal bleeding, or staff concern regarding an individual’s condition. When cardiopulmonary arrest or another immediately life-threatening condition was identified during an RRS response, resuscitative measures, including cardiopulmonary resuscitation, defibrillation, airway support, and transfer to the emergency resuscitation pathway, were initiated as part of the RRS response.

After activation, the 24-h monitoring center immediately broadcast the event location and code. During nighttime hours (19:00–07:00 the next day), team members are notified directly by telephone. Team members carry portable emergency equipment and are required to arrive on site within 5 min. Standardized emergency equipment, including resuscitation carts, defibrillators, and emergency kits, is uniformly deployed throughout the hospital.

### Intelligent RRS information management platform

The intelligent RRS management platform was developed within the hospital information system (HIS) architecture and integrates emergency activation, mobile terminal-based data entry, structured databases, and visual monitoring modules. The electronic event registration form was an existing institutional module embedded in the platform for routine RRS documentation and quality management. Once an event is triggered, relevant information is entered into the system in real time or immediately after on-site management via mobile devices and automatically synchronized to the server to generate an emergency event database. Recorded information includes event location, trigger reason, activator identity, response interval, on-site assessment, emergency interventions, and patient disposition. A visual dashboard displays event location, response time, interventions, and patient outcomes, thereby enabling process monitoring and quality tracking.

The platform also supports historical data mining and statistical analysis, which can be used to identify high-risk areas, optimize zone assignments, and improve emergency workflows. In addition, the platform has the potential to interface with other hospital information systems, including emergency triage, laboratory testing, medical imaging, surgery and anesthesia, and in-hospital transport systems, thereby supporting future digital integration of emergency care pathways. In this study, the intelligent emergency response information platform was used as a digital workflow management tool. The platform supported standardized event registration, time-stamped documentation, response process monitoring, and retrospective workflow review. It enabled the extraction of key operational variables, including event location, trigger reason, activator identity, response interval, on-site intervention, and transfer destination. These data were used to describe real-world RRS performance and to explore the process management value of hospital-wide emergency response for non-inpatient individuals in hospital public areas.

### Data collection

A standardized electronic emergency event form was used to capture baseline characteristics, event location, activator identity, trigger indications, response efficiency, interventions, and outcomes. After on-site management was completed, the emergency response team verified the event information entered into the electronic platform. All records were synchronized to the hospital data center. Before statistical analysis, the data underwent multistage quality control, including completeness checks, logical validation, and outlier correction.

For individuals transferred to the emergency department, emergency triage levels were assigned according to the Chinese Expert Consensus on Emergency Pre-examination and Triage (11), which uses a four-level triage system based on urgency and severity. Level I indicates a critically ill condition requiring immediate resuscitation, level II indicates an emergent condition requiring rapid assessment and treatment, level III indicates an urgent condition requiring timely emergency care, and level IV indicates a less urgent or non-urgent condition.

For the exploratory analysis, high-risk events were operationally defined as RRS activations that required subsequent management in the emergency resuscitation room after initial on-site assessment and stabilization. Non-high-risk events were defined as activations that did not require emergency resuscitation room management and were managed through emergency department evaluation, brief observation, outpatient follow-up, or other non-resuscitation pathways.

### Statistical analysis

Because this was a retrospective complete-enumeration study of all eligible RRS activations during the study period, no a priori sample size calculation was performed. The final sample size was determined by the number of eligible events recorded in the institutional RRS registry between January 2015 and December 2025. This sample was considered adequate for descriptive analysis of RRS activation characteristics, whereas exploratory analyses of factors associated with high-risk events were considered hypothesis-generating rather than confirmatory because of the modest sample size, absence of multivariable adjustment, and risk of sparse-cell bias.

Statistical analyses were performed using SPSS version 28.0. Continuous variables are presented as median and interquartile range (IQR) and were compared using non-parametric tests. Categorical variables are presented as frequencies and percentages and were compared using the chi-square test or Fisher’s exact test, as appropriate. Descriptive analyses were performed to summarize the incidence and distribution of activations, clinical and operational characteristics of the activation process, activator identity, response time, on-site interventions, and patient disposition. For exploratory univariate analyses, event severity (high-risk vs. non-high-risk events) was used as the dependent variable, and age, time period, trigger location, and trigger reason were examined as independent variables. A two-sided *P* < 0.05 was considered statistically significant.

## Results

### General characteristics of the study population

A total of 217 eligible RRS activations were included. The study population showed a slight male predominance and a median age of 52 years (Table 1). Most cases involved non-inpatient individuals who had come to the hospital for medical care, whereas only a small proportion involved staff or volunteers.

**TABLE 1:** Demographic characteristics of the study population (*n* = 217).

Category	Cases (*n*)	%
Sex		
Male	121	55.76
Female	96	44.24
Type		
Non-inpatient individual	213	98.16
Staff	3	1.38
Volunteer	1	0.46
Age group		
0–17	13	5.99
18–44	71	32.72
45–59	44	20.28
60–74	52	23.96
75–89	29	13.36
Unknown	8	3.69

Age categories were defined according to the observed age distribution of the study cohort to provide descriptive strata with relatively balanced cell sizes. These categories were used for descriptive and exploratory purposes only and do not represent established clinical risk thresholds.

### Reasons for RRS activation

The reasons for activation are summarized in Table 2. Sudden loss of consciousness, witnessed collapse, and syncope/dizziness accounted for most calls.

**TABLE 2:** Distribution of RRS trigger reasons (*n* = 217).

Trigger reason	Cases (*n*)	%
Unspecified sudden loss of consciousness	77	35.48
Witnessed sudden collapse	35	16.13
Syncope/presyncope or dizziness	34	15.67
Staff concern regarding individual condition	29	13.36
Convulsion/epileptic seizure	16	7.37
Drug allergy (contrast agent, fluorescein, etc.)	10	4.61
Respiratory distress	8	3.69
Vasovagal reaction to blood	6	2.76
Hematemesis/gastrointestinal bleeding	2	0.92

Trigger reason refers to the primary clinical presentation recorded at the time of RRS activation rather than the final diagnosis. “Unspecified sudden loss of consciousness” was used when acute loss of consciousness was recorded but the specific type could not be further classified from the activation record.

### Temporal distribution of RRS activations

**FIGURE 1:**
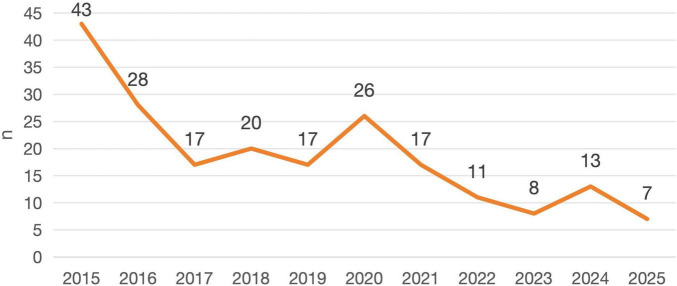
Annual number of RRS activations from 2015 to 2025.

**FIGURE 2:**
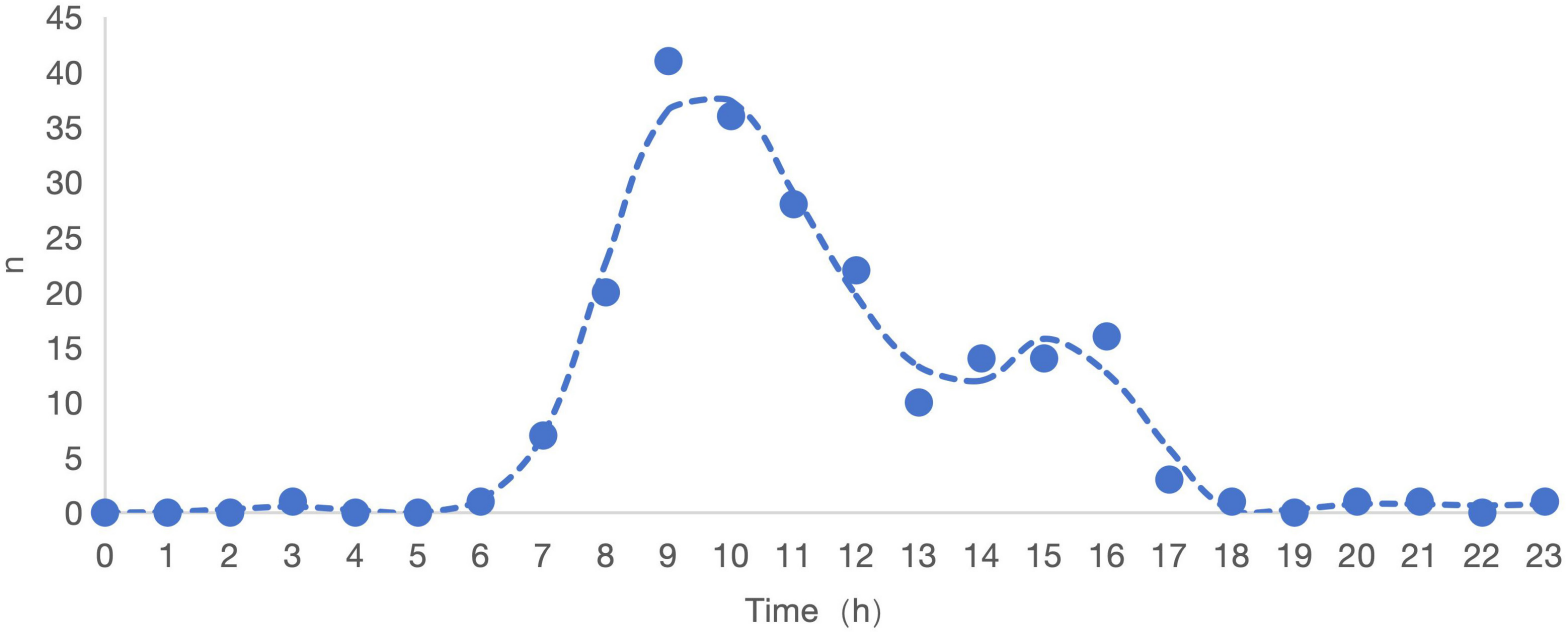
Twenty-four-hour distribution of RRS activations.

Activations clustered in waiting areas, outpatient clinical areas, and medical examination areas and occurred predominantly during weekday daytime hours (Tables 3–5 and Figures 1, 2). Nurses were the most frequent activators (Table 4).

**TABLE 3:** Temporal distribution of RRS activations (*n* = 217).

Time variable	Cases (*n*)	%
Day type:		
Weekday	182	83.87
Weekend/public holiday	35	16.13
Calendar quarter:		
First quarter	71	32.72
Second quarter	54	24.88
Third quarter	43	19.82
Fourth quarter	49	22.58
Time of day:		
08:00-16:00	176	81.11
16:00–24:00	20	9.22
00:00–08:00	21	9.68

First quarter refers to January to March, second quarter to April to June, third quarter to July to September, and fourth quarter to October to December. Weekday refers to Monday to Friday, excluding public holidays.

**TABLE 4:** Distribution of personnel initiating RRS activation (*n* = 217).

Activator	Cases (*n*)	%
Nurse	134	61.75
Physician	37	17.05
Administrative	14	6.45
Security staff	13	5.99
Medical technician	12	5.53
Worker	2	0.92
Volunteer	2	0.92
Unknown	3	1.38

**TABLE 5:** Distribution of RRS activation locations (*n* = 217).

Location category	*n* (%)	Location category	*n* (%)
**Clinical care areas**	64 (29.49)	**Public open areas**	109 (50.23)
Ophthalmology	35 (54.69)	Waiting area	96 (88.07)
Cardiology	12 (18.75)	Hospital entrance	6 (5.50)
Infusion room	6 (9.38)	Toilet/hot water room	3 (2.75)
Respiratory clinic	3 (4.69)	Garden	2 (1.83)
**Traditional Chinese Medicine Clinic**	3 (4.69)	Skybridge	1 (0.92)
Neurology	2 (3.13)	Parking garage	1 (0.92)
Otolaryngology	2 (3.13)		
Dentistry	1 (1.56)		
**Medical examination areas**	40 (18.43)	**Administrative areas**	4 (1.84)
Blood collection/laboratory	25 (62.50)	Administrative office	3 (75.00)
ECG/EEG room	6 (15.0)	Staff cafeteria	1 (25.00)
CT/ECT/MRI room	5 (12.50)		
Ultrasound room	3 (7.50)		
Pulmonary function room	1 (2.50)		

### Response time of the RRS

The overall median response time was 3.00 min. Longer response times were observed in administrative areas, although the number of events in these areas was small (Table 6). Primary on-site interventions were dominated by basic supportive and resuscitative measures. Seven activations had no active on-site intervention recorded (Table 7). After RRS activation, 193 individuals (88.94%) were transferred to the emergency department for further evaluation and treatment (Table 8).

**TABLE 6:** Response time distribution following RRS activation (*n* = 217).

Variable/category	Response time, min, median (P25, P75)	Test statistic	*p*
Overall (*n* = 217)	3.00 (2.00,4.00)	–	–
Location category		*H* = 10.782	0.013
Clinical care areas (*n* = 64)	3.00 (2.00, 4.00)		
Medical examination areas (*n* = 40)	3.00 (2.00, 4.00)		
Public open areas (*n* = 109)	3.00 (2.00, 4.00)		
Administrative areas (*n* = 4)	5.50 (5.00, 6.50)		
Day type		*Z* = 1.284	0.199
Weekday (*n* = 182)	3.00 (2.00, 4.00)		
Weekend/public holiday (*n* = 35)	3.00 (1.50, 4.00)		
Time of day		*H* = 4.450	0.108
08:00∼16:00 (*n* = 176)	3.00 (2.00, 4.00)		
16:00∼24:00 (*n* = 20)	2.00 (1.00, 3.00)		
00:00∼08:00 (*n* = 21)	3.00 (2.00, 4.00)		

*P* < 0.05 indicates a statistically significant difference. The longer response time in administrative/logistics areas should be interpreted in light of the small number of events in these areas.

**TABLE 7:** Distribution of on-site interventions (*n* = 217).

Intervention	Frequency(n)	%
Oxygen therapy	56	25.81
Blood glucose monitoring	55	25.35
Cardiopulmonary resuscitation	25	11.52
Intravenous medication	23	10.60
Bag-mask ventilation	20	9.22
Electrocardiography	18	8.29
Endotracheal intubation	8	3.69
Defibrillation	4	1.84
Suctioning	1	0.46
No on-site intervention	7	3.23

When multiple interventions were provided during the same activation, the main or most clinically relevant intervention was used for classification.

**TABLE 8:** Outcomes after RRS activation (*n* = 217).

Outcome	Cases (*n*)	%
Immediate disposition after RRS activation		
Transferred to ER	193	88.94
Not transferred to ER	24	11.06
Subsequent outcome transferred to ER department		
Discharged after ER evaluation	139	72.02
Hospitalized	46	23.83
ICU	3	1.55
Death	5	2.59
Left voluntarily	6	25.00
Discharged after brief intervention	17	70.83
Underwent surgery/intervention	1	4.17

ER, Emergency Department.

### Factors associated with high-risk events

Exploratory univariate analyses suggested that age and trigger reason were associated with event severity, whereas time period and trigger location were not (Table 9).

**TABLE 9:** Univariate analysis of factors associated with high-risk events.

Variable	High-risk events	Non-high-risk events	Statistic	*p*
Age[P_50_ (P_25_, P_75_), (*n* = 209)]	56.00 (33.00, 67.25)	49.00 (29.00, 60.00)	−2.320^1^	0.021
Age[n,(%), (*n* = 209)]	5 (4.17%)	8 (8.99%)	8.266^2^	0.082
0–17				
18–44	40 (33.33%)	31 (34.83%)		
45–59	20 (16.67%)	24 (26.97%)		
60–74	36 (30.00%)	16 (17.98%)		
75–89	19 (15.83%)	10 (11.24%)		
Time period			1.385^2^	0.500
Trigger location			–	0.303
Trigger reason			–	0.002

Age data were available for 209 activations; age information was missing in eight activations.

^1^*Z* value from the Mann–Whitney U test;

^2^chi-square value from Pearson’s chi-square test.

## Discussion

### Principal findings

This retrospective study describes the operational characteristics of an in-hospital RRS for non-inpatient individuals in hospital public areas. The median response time was 3.00 min, nearly 90% of individuals required transfer to the emergency department, and common interventions included oxygen therapy, blood glucose testing, and basic resuscitative measures. These findings suggest that public-area emergencies are clinically important, operationally diverse, and highly relevant to hospital safety governance.

### Importance of implementing an RRS in public areas

Most existing studies and applications of RRS focus on early warning and intervention for deteriorating hospitalized patients and have reported favorable outcomes (12–15). In contrast, relatively few studies have specifically addressed sudden medical events among non-inpatient individuals in hospital public areas (16). In the present study, 88.94% of individuals were transferred to the emergency department for further treatment, and a substantial proportion of those with available triage data were assigned to critical triage categories. These findings suggest that emergency events involving non-inpatient individuals in hospital public areas may represent clinically meaningful safety risks and require an organized hospital-wide response mechanism. The establishment of an RRS for public-area emergencies is also consistent with national policy requirements for strengthening emergency care quality and patient safety. The 2024 national emergency medicine quality control indicators emphasize standardized quality management and continuous improvement in emergency care (17). In addition, national medical quality and patient safety initiatives for 2023–2025 highlight the need to optimize outpatient and emergency care workflows, strengthen safety management across healthcare processes, and identify risks within the hospital environment (18). Therefore, extending an RRS beyond inpatient wards may help hospitals translate these requirements into an operational mechanism for early recognition, rapid response, and safe transition of care for non-inpatient individuals in public areas.

### Need for continuous emergency coverage across all hospital areas

In this study, the 217 RRS events occurring in hospital public areas showed clear temporal and spatial heterogeneity. Waiting areas (44.24%), outpatient clinic areas (29.49%), and medical examination areas (18.43%) constituted the three major high-incidence zones. In addition, events were observed in nontraditional healthcare spaces such as toilets, elevators, outdoor gardens, and underground parking areas.

Notably, 18.89% of RRS activations occurred between 16:00 and 08:00, which is higher than the proportion of nighttime emergency events reported in a hospital in Barcelona, Spain (15.2%) (19). This indicates that even outside regular outpatient service hours, a substantial number of non-inpatient individuals remain in hospital public areas and are mobile and unmonitored, thereby increasing the likelihood of sudden medical events. The hospital-wide emergency call and response system, with full spatial coverage and seamless connection to outpatient services, enables response performance during nighttime and holidays comparable to that during regular working hours.

The association between older age, certain trigger reasons, and high-risk events is clinically plausible and consistent with previous RRS literature. Older individuals may have reduced physiological reserve, multiple comorbidities, and atypical presentations, which can increase the risk of rapid deterioration after an acute event. Previous studies of team-based rapid response models have shown that individuals reviewed for respiratory distress or hypotension are often elderly and have substantial mortality, and that abnormal vital signs can predict subsequent rapid response activation after emergency department admission (20, 21). Therefore, in public hospital areas where continuous monitoring is absent, older age and high-acuity presentations, such as sudden loss of consciousness, seizure-like activity, respiratory distress, or gastrointestinal bleeding, may serve as practical warning signals for early escalation and rapid transfer to definitive emergency care. The lack of clear restriction of high-risk events to specific locations or time periods further supports the need for an all-area, all-time rapid response mechanism.

### Continuous optimization through data-driven and precision-oriented decision-making

In-hospital emergency response depends not only on the clinical competence of response team members but also on the spatial organization of response coverage and the ability to monitor process indicators over time. Previous RRS reporting and quality-improvement frameworks have emphasized the importance of process measures such as activation characteristics, response intervals, reasons for activation, interventions, and subsequent patient disposition when evaluating system performance (22). In the present study, the median response time was 3.00 min, which was within the institutional target of arrival within 5 min. This finding suggests that a responsibility-zone model based on building distribution and shortest travel distance may support timely access to emergency care for non-inpatient individuals in hospital public areas.

However, the variation in response time across hospital areas also indicates that RRS performance is influenced by local spatial layout and accessibility. Longer response intervals in administrative or non-clinical areas, although based on small event numbers, suggest that emergency preparedness should not be limited to traditional clinical spaces. Continuous monitoring of response intervals and event locations may help hospitals identify areas with delayed access, adjust zone assignments, optimize emergency equipment deployment, and improve route planning. These findings are consistent with the broader view that an RRS should be evaluated not only by clinical outcomes but also by whether the system functions as intended across different locations and time periods (23).

The distribution of interventions further suggests that many public-area emergencies require rapid basic stabilization before definitive emergency department care (24). In this study, the most frequent interventions included oxygen therapy, blood glucose monitoring, cardiopulmonary resuscitation, intravenous medication administration, and bag-mask ventilation. More than 80% of core interventions were within the scope of basic life support or early emergency stabilization, indicating that standardized training in high-frequency skills, clear role allocation, and readily available emergency equipment may be particularly important for hospital-wide RRS preparedness. Therefore, structured analysis of RRS activation data can support targeted improvements in coverage design, staff training, and emergency workflow management for non-inpatient individuals in public hospital areas.

### Process-management implications of structured RRS documentation

The findings of this study highlight the process management value of digitally recorded RRS activations in hospital public areas. Unlike inpatient deterioration, which is usually embedded within a continuous monitoring and nursing responsibility system, public-area emergencies require rapid recognition, responsibility assignment, team dispatch, on-site stabilization, and transfer coordination across multiple hospital spaces. Digital capture of event location, trigger reason, activator identity, response interval, intervention, and disposition provides a structured basis for reviewing these processes and identifying operational bottlenecks (25). In this study, the information platform did not function as a newly developed predictive model or artificial intelligence algorithm; rather, it served as a digital infrastructure for emergency workflow documentation and quality improvement. Such structured process data may support future refinement of hospital-wide emergency response pathways and provide a practical foundation for subsequent digitally integrated emergency management systems.

### Strengths and limitations

This study provides a relatively long observation period and reflects the real-world operation of a hospital-wide RRS for non-inpatient individuals, a setting that remains underreported in the literature. Nevertheless, several limitations should be acknowledged. First, this was a single-center retrospective study with a modest sample size, which limits external validity. Second, the analysis was primarily descriptive and exploratory, without multivariable adjustment; therefore, findings regarding factors associated with high-risk events should be interpreted cautiously because of potential confounding and sparse-cell bias. Third, event classification and clinical documentation were based on routine operational records and may be subject to information bias or misclassification. The operational category of “non-inpatient individuals” may also have included heterogeneous groups, including some individuals awaiting formal assessment in outpatient or emergency areas. Finally, this study did not include a pre-implementation comparison group or denominator data, such as annual outpatient volume, visitor volume, staff flow, or public-area occupancy. Therefore, causal inference regarding RRS effectiveness cannot be made, and the apparent decrease in annual RRS activations over time should be interpreted cautiously. Future studies should incorporate denominator data and information on operational changes to evaluate temporal trends in public-area RRS activations more rigorously.

## Conclusion

The in-hospital RRS in this study supported timely recognition and initial management of emergency events involving non-inpatient individuals in hospital public areas. These events represent a distinct operational scenario characterized by the absence of continuous monitoring, heterogeneous activators, spatial dispersion, and the need for rapid hospital-wide coordination. Most affected individuals required further emergency department care, and older age and trigger reason were associated with a higher likelihood of high-risk events. Spatially distributed coverage, standardized response workflows, and digital process monitoring may strengthen emergency preparedness and workflow management for non-inpatient individuals in hospital public areas. Future optimization of digital emergency response systems should further enhance standardized electronic registration, time-stamped process monitoring, linkage with emergency department triage and transfer pathways, and multicenter evaluation of clinical and operational outcomes.

## Data Availability

The original contributions presented in this study are included in this article/supplementary material, further inquiries can be directed to the corresponding authors.
